# The prevalence of Fabry disease in a statewide chronic kidney disease cohort – Outcomes of the aCQuiRE (Ckd.Qld fabRy Epidemiology) study

**DOI:** 10.1186/s12882-022-02805-8

**Published:** 2022-05-04

**Authors:** Andrew Mallett, Phoebe Jane Kearey, Anne Cameron, Helen G. Healy, Charles Denaro, Mark Thomas, Vincent W. Lee, Samantha Louise Stark, Maria Fuller, Zaimin Wang, Wendy E. Hoy

**Affiliations:** 1 Department of Renal Medicine, Townsville University Hospital, Townsville, Australia; 2 College of Medicine and Dentistry, James Cook University, Townsville, Australia; 3 Institute for Molecular Bioscience, The University of Queensland, Brisbane, Australia; 4 Faculty of Medicine, The University of Queensland, Brisbane, Australia; 5 CKD.QLD and NHMRC CKD.CRE, The University of Queensland, Brisbane, Australia; 6 Kidney Health Service, Royal Brisbane and Women’s Hospital, Brisbane, Australia; 7 Department of Internal Medicine and Aged Care, Royal Brisbane and Women’s Hospital, Brisbane, Australia; 8 Department of Nephrology, Royal Perth Hospital, Perth, Australia; 9 Department of Renal Medicine, Westmead Hospital, Sydney, Australia; 10 Sydney Medical School, Faculty of Medicine and Health, University of Sydney, Sydney, Australia; 11 Genetics and Molecular Pathology Laboratory (SA Pathology), Adelaide, Australia; 12 Adelaide Medical School, University of Adelaide, Adelaide, Australia

**Keywords:** Fabry disease, Screening, Chronic kidney disease

## Abstract

**Background:**

Prevalence of Fabry disease amongst Chronic Kidney Disease (CKD) patients on haemodialysis has been shown to be approximately 0.2%.

**Methods:**

We undertook a cross-sectional study employing a cascade screening strategy for Fabry Disease amongst 3000 adult, male and female patients affected by CKD stage 1-5D/T at public, specialty renal practices within participating Queensland Hospital and Health Services from October 2017 to August 2019. A multi-tiered FD screening strategy, utilising a combination of dried blood spot (DBS) enzymatic testing, and if low, then lyso-GB3 testing and DNA sequencing, was used.

**Results:**

Mean (SD) age was 64.0 (15.8) years (*n* = 2992), and 57.9% were male. Eight participants withrew out of the 3000 who consented. Of 2992 screened, 6 (0.20%) received a diagnosis of FD, 2902 (96.99%) did not have FD, and 84 (2.81%) received inconclusive results. Of the patients diagnosed with FD, mean age was 48.5 years; 5 were male (0.29%) and 1 was female (0.08%); 4 were on kidney replacement therapy (2 dialysis and 2 transplant); 3 were new diagnoses.

**Conclusions:**

Estimated overall FD prevalence was 0.20%. Screening of the broader CKD population may be beneficial in identifying cases of FD.

**Trial registration:**

The aCQuiRE Study has been prospectively registered with the Queensland Health Database of Research Activity (DORA, https://dora.health.qld.gov.au) as pj09946 (Registered 3rd July 2017).

**Supplementary Information:**

The online version contains supplementary material available at 10.1186/s12882-022-02805-8.

## Background

Fabry Disease (FD) is a rare genetic condition caused by the absence or deficiency of the enzyme alpha-galactosidase A (α-Gal A), which leads to build up of glycosphingolipids, in particular globotriaosylceramide (GB3), throughout the body in a variety of cell types and tissues [1]. FD affects multiple systems in the body and symptoms and signs vary in type and severity amongst patients. Major causes of morbidity and mortality amongst patients affected by FD include renal, cardiac and cerebrovascular manifestations [2–4]. Other affected systems include skin, ocular, nervous, gastrointestinal, respiratory, lymphatic and skeletal (1). FD has also been found to be associated with poorer quality of life (QOL) [5–7]. Although an X-linked disorder affecting the GLA gene [8, 9], females have also been shown to be affected [10–12], with 69.4 to 80.0% of affected females reporting some degree of symptoms [13, 14] with greater variability of severity and age at onset compared to males with FD [15].

Overall population prevalence of FD is estimated at 0.04% (range = 0.0 to 0.4%) [16]. Large scale screening studies of neonates have estimated prevalence at between 0.013% [17] and 0.05% [18] for males and 0.0% [18] and 0.002% [19] for females, though some populations experience increased prevalence of particular disease-causing alleles sich as Taiwan. Overall prevalence of FD in haemodialysis populations world-wide is estimated to be 0.12% [20, 21] to 0.36% [22]. In a systematic review of FD screening in dialysis populations, Linthorst, et al. [23], estimated prevalence at 0.33% for males and 0.1% for females. Doheney, et al. [24], conducted a reanalysis of 63 studies that estimated prevalence of FD in haemodialysis, kidney transplant, stroke & cardiac populations with GLA mutations (total *n* = 36,820 patients; 23,954 males, 12,866 females). Revised prevalence estimate for haemodialysis patients was 0.21% in males and 0.15% in females. Revised prevalence rates for kidney transplant patients based upon α-Gal A enzymatic screening was 0.24% in males and 0.00% in females.

The prevalence of FD within the broader CKD population is less well studied. Two Turkish studies of pre-terminal CKD patients (i.e. patients with CKD stages 1 through 5 [25] and not on kidney replacement therapy (KRT) identified FD in 1.8% [26] and 0.4% [27] male patients but not in female patients [26, 27]. Favalli, et al. [28], examined the prevalence of FD in patients selected from multiple settings and also identified FD in male patients (2.7%) but not female patients. Sample sizes for these studies ranged from 72 [28] to 1453 [27]. Lack of identified cases in female patients may be due to true lack of undiagnosed female FD cases, less sensitive screening methods, or insufficient sample size.

FD can be identified through screening, including Dried Blood Spot (DBS) testing to analyse blood levels of the α-galactosidase A enzyme, lyso-GB3 testing and genetic testing [29]. Whilst DBS-based non-genetic screening is imperfect amongst women, the feasibility of a direct genetic sequencing screening approach is not feasible in current local operational settings. As an X-linked disorder with variable phenotype amongst women, we anticipate that there might be two genetically affected women in the broader community for every male identified. Several treatments are available for FD, including Enzyme Replacement Therapy (ERT). Two preparations of ERT shown to stabilise or improve FD symptomatology [30, 31] are currently funded under the Life Saving Drugs Program in Australia for FD patients.

The Ckd. Qld fabRy Epidemiology (aCQuiRE) study [32] targeted a significantly larger screening sample of 3000 CKD patients. We aimed to identify FD in patients with CKD in public, specialty nephrology practices.

## Methods

The detailed aCQuiRE study protocol has been previously published [32].

### Patients

A sample size of 3000 participants was chosen to maximise the likelihood of detecting FD disease in this CKD patient population based on an estimated prevalence of FD disease amongst CKD patients of 0.0–1.5% (working assumption of 0.2%). To be eligible to participate, patients needed to: be adult, aged 18 years and over; be under the care of a renal specialist within Queensland Health, the state public universal healthcare department; have chronic kidney disease stage 1-5D/T; be able to provide informed consent; be a patient at an approved and participating site; and be eligible for Medicare, the national public universal health service funding agency in Australia. The patient information and consent form included information about FD including its genetic basis and aetiology, consequences and potential therapeutic implications.

### Sites

Three thousand adult male and female patients affected by stage 1-5D/T CKD under clinical care were screened at their treating clinical service at seven metropolitan and regional sites: Royal Brisbane and Women’s Hospital (RBWH); Cairns Hospital; Logan Hospital; Toowoomba Hospital; Mackay Base Hospital; Hervey Bay Hospital; and Gold Coast University Hospital. Sites received a site establishment fee of AUD$7000 and reimbursement for nursing time involved in screening patients.

### Screening, diagnosis and referral

The aCQuiRE Study employed a within-patient, cascade screening strategy with sequential laboratory testing comprising a combination of dried blood spot (DBS) α-Gal A testing, lyso-GB3 testing and DNA sequencing. First-Patient-In was screened during October 2017 and Last-Patient-In was screened during August 2019. For patients who received test results outside the reference range, Females were offered follow-up lyso-GB3 testing concurrent with genetic testing and Males had follow-up confirmatory genetic testing. Where a result indicated a potential sample quality issue, patients were asked to undergo repeat DBS testing and/or lyso-GB3 testing. Diagnoses were confirmed through sequencing of the GLA gene reporting a detection rate of 95%. For patients who had a previous diagnosis of FD, diagnosis was confirmed through plasma lyso-GB3 determinations.

### Dried blood spot (DBS) α-Gal a

DBS samples were collected by local clinical staff using the finger prick method prior to dialysis and heparinisation. This sample acquisition method is in keeping with operational learnings from a previous Australian FD screening study [33]. A control enzyme (beta-galactosidase) was also measured to ensure integrity of the blood spots. A repeat DBS test was conducted for patients with a reported sample quality issue.

### Lyso-GB3 testing

Patients who required follow-up lyso-GB3 testing had blood collected at their on-site Pathology Queensland laboratory. Frozen plasma samples were batched and sent to the Genetics and Molecular Pathology Laboratory (SA Pathology) for analysis as described previously [29, 34].

### Genetic testing

Where genetic testing was indicated, patients had blood collected at the on-site Pathology Queensland laboratory. Extracted DNA was sent to the Genetics and Molecular Pathology Laboratory (SA Pathology) for clinically accredited *GLA* Sanger sequencing, analysis and reporting. A diagnosis of FD was defined as a patient found to have a pathogenic or likely pathogenic *GLA* result, either preceding the study (previous diagnosis of FD) or as a result of genetic testing indicated by the study screening strategy (new diagnosis of FD).

The treating physician informed the patient where pathology results indicated FD. Patients with newly identified cases of FD were offered referral to the Queensland Statewide FD Treatment Service (QSFTS) for confirmation of their diagnosis and potential ERT treatment. Genetic counselling and FD screening was also offered to FD patients and their at-risk family members.

### Data capture

Three case report forms were used to capture patient demographics, relevant medical history and to track screening, diagnosis and referral. Data was entered into a central online database using REDCap electronic data capture tools [35] hosted at The University of Queensland.

## Results

**Fig. 1:**
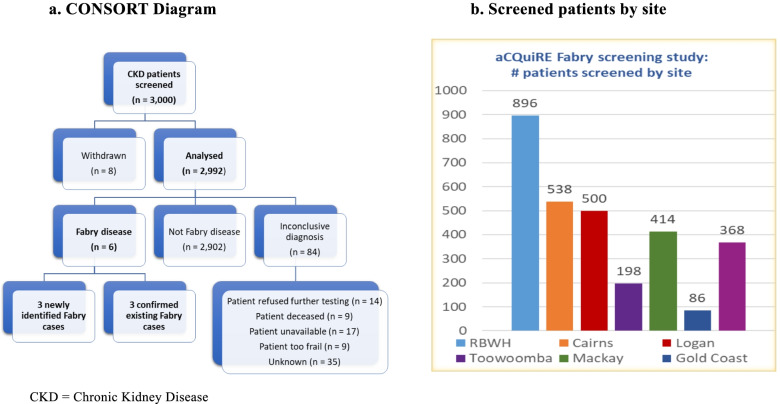
Screened patients

A total of 3000 CKD patients were screened across seven sites. Eight patients withdrew consent after screening. Of the remaining 2992 screened patients, six (0.20%) patients were diagnosed with FD (Supplementary Table 1), 2902 (96.99%) patients did not have FD, and 84 (2.81%) patients received inconclusive results (See Fig. 1).

### Identified cases of Fabry disease

Of the six confirmed FD cases that were identified, three had previously been diagnosed with FD and three were newly identified through the aCQuiRE Study (see Supplementary Table 1). All three of the newly diagnosed FD cases had progressed to kidney failure (KF) at the time they underwent screening as part of their study participation. Five patients were male (0.30%) and one patient was female (0.08%). Collectively, they had 28 family members potentially at-risk of FD, ranging from four to seven people. The outcomes of subsequent screening of these at-risk family members is not known as they were not participants in the study, however all have been informed by their affected family member and offered potential referral for counselling and screening from the QSFTS.

### Study cohort characteristics

Mean age at enrolment was 64.0 years (SD = 15.8, *n* = 2992) and 57.9% were male. Age at consent is shown in Supplementary Fig. 1, in summary statistics and in distribution as dotplots. The FD patients were significantly younger at screening than the other screened CKD patients (Supplementary Table 2). Age at consent was 48.5 years for patients with FD compared to 63.9 years for patients without FD (*p* = 0.0313).

Of the screened CKD patients, 6.2% identified as Aboriginal and/or Torres Strait Islander, which is slightly higher than the state average of 4.6% [36], consistent with indigenous populations having higher rates of CKD compared to the general population [37].

Approximately one quarter (25.4%) of CKD patients were receiving KRT, i.e. dialysis (Stage 5D) or transplant (Stage 5 T), with the remaining 74.6% of patients split across the pre-KRT stages (Supplementary Fig. 2). As a pragmatic screening study, sites screened between 31.0 and 60.0% of the CKD patient population at their site.

Overall, 12 study participants (0.4%) reported a family history of FD: eight men (0.5%) and four women (0.3%). The sole female patient identified as having FD reported a family history of both CKD and FD. None of the identified male patients affected by FD reported a history of CKD or FD.

### Inconclusive results and participant Withdrawls

Reasons for inconclusive results included that the patient opted out of follow-up testing, died before testing was finalised, or was unavailable or too frail to complete follow-up testing. The treating physician followed up with any patients who they perceived had a clinical reason to rule out FD. Patients with inconclusive results had either marginally low or high alpha-Gal A results that were likely to be due to sample quality issues.

The average age at consent of patients who withdrew was 65.4 years, with an equal number of males and females (4 males, 4 females). The ethical approval of the study precluded an ability to record the number of patients offered participation but who declined prior to providing consent.

## Discussion

Six cases of FD were identified among 2992 adult, male and female CKD patients. This is an overall estimated FD prevalence of 0.20%, which is consistent with previously published estimates for dialysis populations and a working hypothesis of 0.2%. Twenty-eight at-risk family members were identified (4–7/FD case identified) who would benefit from family screening.

Of the six CKD patients diagnosed with FD, 5 were male (0.29%) and 1 was female (0.8%). The identification of a case in a female patient highlights the importance of including women in a FD screening program to enable a true estimate of prevalence and for targeted treatment and management. Three of the six cases of FD had been previously identified with all three receiving enzyme replacement therapy. Exclusion of these previously identified cases would have potentially underestimated prevalence.

An upper age limit was not stipulated and all FD cases in the adult CKD population had the possibility of being included. The ages of patients diagnosed with FD ranged from 23 to 70 years, indicating FD as a potential causative factor for CKD patients of all ages. The CKD patients diagnosed with FD were significantly younger than the CKD patients without FD, consistent with persons with genetically driven kidney disease and those with glomerulonephritis in the broader adult CKD.QLD population having a younger age at diagnosis than those with CKD due to diabetes and renovascular disease (42).

FD cases were identified in patients with early through to late-stage kidney disease, though all newly identified cases had already progressed to KF and were receiving KRT. While four patients with FD were receiving KRT, two patients were in earlier stages (Stage 1, Stage 3B), when ERT may be of most benefit [38]. Therefore, this study supports a potential benefit to screening patients in all stages of CKD. No particular pattern of symptoms was identified. This may be due to the small number of cases identified, however is consistent with research that indicates symptom presentation varies among patients [1]. Widespread screening of CKD patients for FD may be beneficial.

Genetic testing is more sensitive for diagnosis of FD but much more costly and time-consuming compared to DBS testing and was not feasible for a large-scale pragmatic screening study such as aCQuiRE. DBS testing of α-Gal A was chosen given cost, time and efficiency. Some issues with the quality of DBS testing were experienced for a proportion of patients. Given that there are known challenges with sole use of α-Gal A testing amongst potentially heterozygous women, this is likely to have impacted underestimation of prevalence amongst females. Other limitations of this study include that 2.81% of participants had an unresolved inconclusive result, that in-depth biochemical data collection from clinial pathology results was not performed, and that individual family pedigrees were not recorded for participants.

Prospective learnings from other FD screening studies were integrated, including sample collection pre-heparinisation for participants receiving haemodialysis [33]. Where still required, cascade testing, including a repeat DBS sample and/or plasma lyso-GB3, resolved sampling issues. There are additional emerging and novel screening strategies for FD as well, including urinary studies [39]. The anticipated number of genetically affected women likely to be present in the community for every identified affected male is 2:1. As such, in this study there may statistically be 9 such genetically affected women present but not identified by the study screening methodology. This might hypothetically be due to the variable FD phenotype experienced by genetically affected women, resulting in a CKD phenotype that may not have yet or ever become present. Further, all three newly identified FD cases had previously undergone kidney biopsy without their clinical diagnosis being revealed. This highlights the need for close and fortified consideration of electron microscopy or toluene blue staining of semi-thin sections as part of kidney biopsy evaluation.

While it is not possible to make statistical conclusions, given the small number of FD cases identified, our results can inform estimates of cost-effectiveness of screening of patients of all ages in adult CKD populations, patients from all stages of CKD and the inclusion of females in screening programs. Sample quality should be consistently monitored. The Queensland Statewide FD Treatment Service identified 28 at-risk family members. These family members were eligible for screening and genetic counselling, with anecdotal evidence that many accessed this. Benefits of wide-spread screening of CKD populations for FD is likely to extend benefits to wider populations through family screening. Initially however, our findings suggest that for every currently identified patient with FD complicated by CKD, there may be a further FD case affected by CKD that is yet to be diagnosed.

## Supplementary Information

**Additional file 1.**

**Additional file 2.**

**Additional file 3.**

## Data Availability

The datasets generated and/or analysed during the current study are not publicly available due ongoing analyses by the aCQuiRE study investigators but may be available from the corresponding author and aCQuiRE study Investigator Committee upon application and request.

## Abbreviations

aCQuiRE Study: Ckd.Qld fabRy Epidemiology Study
CKD: Chronic Kidney Disease
DBS: Dry Blood Spot
DNA: Deoxyribonucleic Acid
eGFR: Estimated Glomerular Filtration Rate (CKD-EPI)
ERT: Enzyme Replacement Therapy
FD: Fabry Disease
GB3: Globotriaosylceramide
GFR: Glomerular Filtration Rate
KF: Kidney Failure
KRT: Kidney Replacement Therapy
QSFTS: Queensland Statewide Fabry Treatment Service
α-Gal A: Alpha-galactosidase A
