# Supplementary material for: The prevalence of Fabry disease in a statewide chronic kidney disease cohort – Outcomes of the aCQuiRE (Ckd.Qld fabRy Epidemiology) study
Source: BMC Nephrol. 2022 May 4;23:169. doi: 10.1186/s12882-022-02805-8 (PMC9066726; doi:10.1186/s12882-022-02805-8)
Supplement: Supplementary file 1 — Additional file 1. [file 12882_2022_2805_MOESM1_ESM.docx]

**Supplementary Table 1. FD cases: demographic and clinical details**

| **Sex** | **Age at consent, years** | **α-Gal** | **Lyso-GB3** | **Diagnosis, Confirmation and Genetic Findings** | **CKD Stage** | **# At-Risk Relatives** |
| --- | --- | --- | --- | --- | --- | --- |
| **Male** | 23 | 0 | <4 | *GLA*, c.335G>A (p.Arg112His), pathogenic (ACMG Class 5), hemizygous *Newly Identified* | Transplant (5T) | 4 |
|  |  | **Clinical Summary**  Kidney Biopsy: at 15 years (presented with reduced eGFR and moderate proteinuria), report unable to be retrieved, clinical notes indicate focal segmental glomerular sclerosis was reported with electron microscopy not performed, histological diagnosis ascribed as focal segmental glomerulosclerosis  Other renal phenotypes: proteinuria (severe prior to dialysis)  Commenced Kidney Replacement Therapy: 19 years (haemodialysis then deceased donor kidney transplantation at 21 years)  Comorbidities: Depression, osteoporosis  Symptoms: Chest pain | | | | |
| **Male** | 57 | 0.1 | <4 | *GLA*, c.335G>A (p.Arg112His), pathogenic (ACMG Class 5), hemizygous *Newly Identified* | Transplant (5T) | 4 |
|  |  | **Clinical Summary**  Kidney Biopsy: at 49 years (presented with reduced eGFR and moderate proteinuria), 16/20 glomeruli globally sclerosed, 3/20 glomeruli demonstrate segmental sclerosis and hyalinosis, 1/20 glomeruli exhibits segmental intracapillary foam cells, glomerular enlargement, significant (70%) interstitial fibrosis and tubular atrophy, vascular changes present (arteriolosclerosis, arteriolar hyalinosis, arteriosclerosis), electron microscopy not performed, histological diagnosis ascribed as advanced secondary focal segmental glomerulosclerosis due to hypertensive nephrosclerosis  Other renal phenotypes: Proteinuria (severe prior to dialysis)  Commenced Kidney Replacement Therapy: 50 years (haemodialysis then deceased donor kidney transplant at 51 years)  Comorbidities: Hypertension, gout, dyslipidaemia, post-transplant diabetes mellites recurrent skin cancers, rectal carcinoma (complete remission after surgical intervention)  Symptoms: Nil reported | | | | |
| **Male** | 49 | 0 | 160 | *GLA*, c.130T>C (p.Trp44Arg), pathogenic (ACMG Class 5), hemizygous *Newly Identified* | Dialysis (5D) | 4 |
|  |  | **Clinical Summary**  Kidney Biopsy: at 44 years (presented with reduced eGFR and moderate proteinuria), 8/12 glomeruli globally sclerosed, 3/12 glomeruli segmentally sclerosed (2/3 with capsular adhesions, 1/3 with globular hyalinosis and some intraglomerular foam cells). 60% interstitial fibrosis and tubular atrophy, electron microscopy not performed, histological diagnosis ascribed as focal segmental glomerulosclerosis  Other renal phenotypes: Proteinuria (severe prior to dialysis)  Commenced Kidney Replacement Therapy: 48 years (haemodialysis)  Comorbidities: Hypothyroidism  Symptoms: Nil reported | | | | |
| **Male** | 60 | 0 | 25 | *GLA*, c.613C>A (p.Pro205Thr), pathogenic (ACMG Class 5), hemizygous, confirmed biochemically | Stage 1 | 5 |
|  |  | **Clinical Summary**  Kidney Biopsy: at 54 years (FD diagnosis existed pre-biopsy, undertaken as part of assessment for enzyme replacement therapy), glomerular changes of FD (abundant foamy cytoplasm in podocytes), 3/18 glomeruli globally sclerosed, nil focal segmental sclerosis, hypertensive-type vascular changes, no significant tubular atrophy/fibrosis, negative immunofluorescence, electronic microscopy not performed  Other renal phenotypes: proteinuria (mild)  Comorbidities: Ischemic heart disease, Myocardial Infarction,  Symptoms: Hearing impairment  FD Therapy: agalsidase beta, intravenous, fortnightly | | | | |
| **Female** | 70 | 1.3 | 10 | *GLA*, c.1193_1196del (p.Glu398Glyfs*5), pathogenic (ACMG Class 5), heterozygous, confirmed biochemically | Stage 3B | 4 |
|  |  | **Clinical Summary**  Kidney Biopsy: nil  Other renal phenotypes: proteinuria (moderate)  Comorbidities: Coronary artery disease, Pacemaker, Asthma, Transient Ischemic Attack,  Symptoms: Chest pain, Palpitations, Shortness of Breath, Wheeze, Rash, Peripheral Pain, Vertigo, Tinnitus  FD Therapy: agalsidase alfa, intravenous, fortnightly | | | | |
| **Male** | 30 | 0.2 | 50 | *GLA*, c.793C>T (p.Pro265Ser), pathogenic (ACMG Class 5), hemizygous, confirmed biochemically | Dialysis (5D) | 7 |
|  |  | **Clinical Summary**  Kidney Biopsy: at 21years (FD diagnosis made on biopsy, presented with moderate proteinuria and normal kidney function), diffuse podocyte enlargement with foamy cytoplasm, focal foamy changes in parietal cells and some tubular epithelial cells, mild and focal subcapsular tubular atrophy and interstitial fibrosis, enlarged lysosomes within podocytes and “zebra bodies” on electron microscopy  Other renal phenotypes: proteinuria (severe prior to dialysis)  Commenced Kidney Replacement Therapy: 28 years (haemodialysis)  Comorbidities: Nil reported  Symptoms: Peripheral pain, tinnitus, heat intolerance  FD Therapy: agalsidase alfa, intravenous, fortnightly | | | | |

***CKD = Chronic Kidney Disease***

***FD = Fabry Disease***

***ACMG = American College of Genetics and Genomics***

**Supplementary Table 2. Age at consent for FD vs non-FD**

| Fabry diagnosis | N | Mean | sd | P50 Median |  | min | max |
| --- | --- | --- | --- | --- | --- | --- | --- |
| Fabry disease | 6 | 48.5 | 18.3 | 53.2 |  | 23.1 | 70.6 |
| Not Fabry | 2902 | 63.9 | 15.1 | 66.8 |  | 18.2 | 95.2 |
| Total | 2908 | 63.9 | 15.1 | 66.8 |  | 18.2 | 95.2 |

Wilcoxon rank sum test (non parametric), p=0.0313

***FD = Fabry Disease***
