# Supplementary material for: The prevalence of Fabry disease in a statewide chronic kidney disease cohort – Outcomes of the aCQuiRE (Ckd.Qld fabRy Epidemiology) study
Source: BMC Nephrol. 2022 May 4;23:169. doi: 10.1186/s12882-022-02805-8 (PMC9066726; doi:10.1186/s12882-022-02805-8)
Supplement: Supplementary file 3 — Additional file 3. [file 12882_2022_2805_MOESM3_ESM.pdf]

Supplementary Figure 2. Proportions of CKD patients by stages

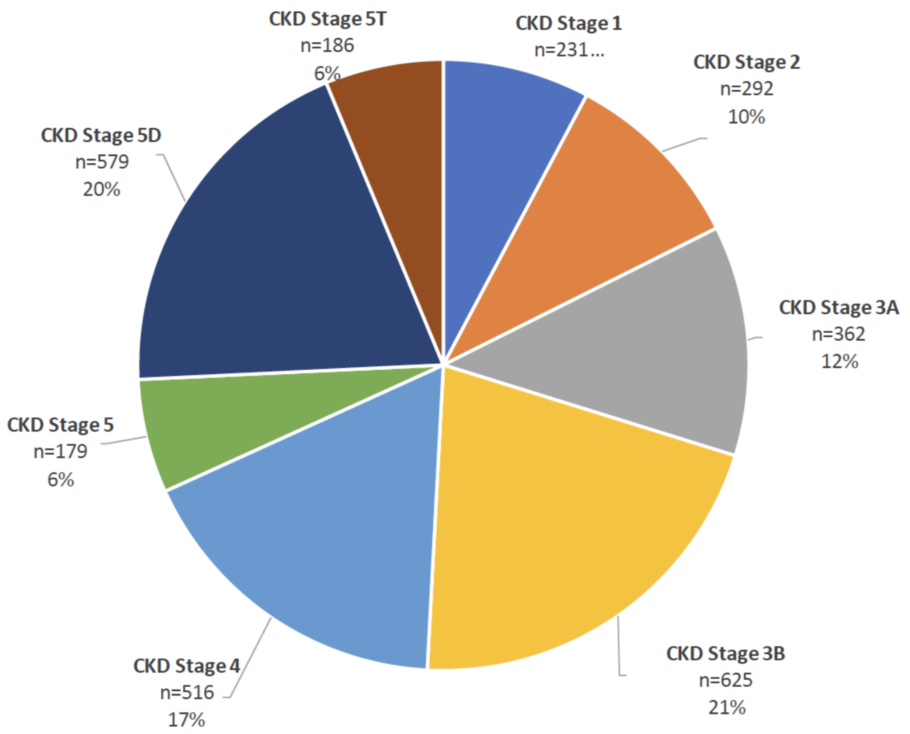

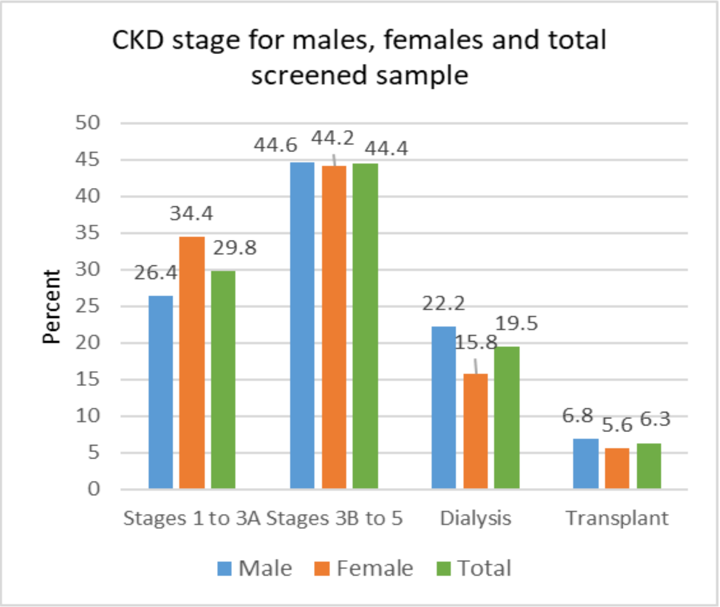

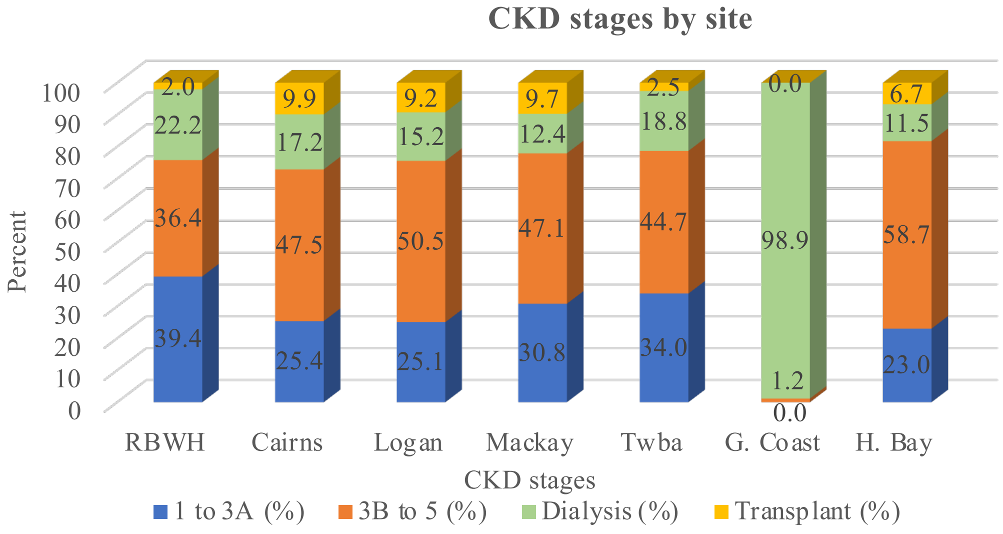

CKD = Chronic Kidney Disease
RBWH = Royal Brisbane and Women's Hospital
Twba = Toowoomba
G.Coast = Gold Coast
H.Bay = Harvey Bay
